# Genetic differences between primary and metastatic cancer: a pan-cancer whole-genome comparison study

**DOI:** 10.1038/s41392-023-01596-0

**Published:** 2023-09-29

**Authors:** Lei Zhong, Zhipeng Zhao, Xiaonan Zhang

**Affiliations:** 1 Department of Pharmacy, Personalized Drug Therapy Key Laboratory of Sichuan Province, Sichuan Provincial People’s Hospital, School of Medicine, University of Electronic Science and Technology of China, Chengdu, Sichuan China; 2 Science for Life Laboratory, Department of Immunology, Genetics and Pathology, Uppsala University, Uppsala, Sweden

**Keywords:** Cancer genomics, Metastasis

Recently, a study by Edwin Cuppen’s group published in *Nature* depicted the genomic differences between late-stage treated metastatic cancers and early-stage untreated primary cancers via a pan-cancer whole-genome sequencing (WGS) analysis.^1^ This study characterizes unique features of metastatic solid tumors and provides a valuable resource for further investigating tumor evolution and treatment resistance.

Activating invasion and metastasis is one of the core hallmarks of cancer,^2^ about 90% of cancer-related death can be attributed to advanced metastatic diseases, and unfortunately, most are incurable by aggressive treatment regimes.^3^ Therefore, it is important to identify genome differences between metastatic and primary tumors and evaluate their influence on treatment resistance. This may help in understanding and leveraging therapeutic interventions, thus to create more effective therapeutic regimens. The study by Edwin Cuppen and colleagues established a harmonized WGS dataset of 7108 tumor samples from 71 cancer types, including more than 4700 metastatic cancer samples from Hartwig Medical Foundation (Hartwig) dataset and more than 2300 untreated primary cancer samples from the Pan-Cancer Analysis of Whole Genomes (PCAWG) Consortium (Fig. 1a). 5365 samples (3451 metastatic and 1914 primary) from 23 cancer types of 14 tissues were finally selected to probe into the genome differences between metastatic and primary tumors (Fig. 1a, b). This is the first time to establish a complete WGS dataset for primary and metastatic cancers using a uniformly processed pipeline and explore the full spectrum of genomic alterations between them.

**Fig. 1:**
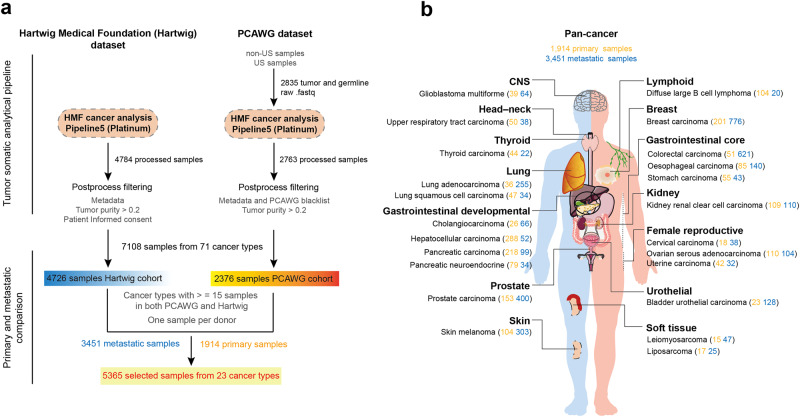
Overview of the cohorts and data processing workflow in this study. **a** Workflow of the harmonized processing pipeline for Hartwig (left) and PCAWG (right) WGS samples. 4784 metastatic cancer samples from Hartwig dataset and 2835 primary tumor samples from PCAWG consortium were all processed using the Hartwig analytical pipeline. Then the output samples were further filtered via a strict quality control. Eventually, 7108 samples from 71 tumor types constitute the unified dataset, and 5365 tumor samples from 23 cancer types were selected to explore genomic differences between primary and metastatic tumors. **b** Anatomical locations of the 23 tumor types included in this study, sorted by tissue or origin. The figure was modified using SMART - Servier Medical Art, provided by Servier, licensed under a Creative Commons Attribution 3.0 Unported License

Through comparison of global genomic features between metastatic and primary cancers, metastatic cancers were found to be generally increased in clonality compared to primary cancers and showed lower intratumor heterogeneity. This may be caused by metastatic events of a subclone originating from the primary tumors or by selective pressure from antitumor treatments.^4^ Further comparison of chromosome arm aneuploidy profiles demonstrated that most metastatic tumors have a conserved karyotype, which is widely acquired at early stages of oncogenesis. Only metastatic thyroid, prostate and clear cell renal cell cancers displayed extensive additional karyotypic changes compared with primary tumors, which is related to the dramatical increases in genomic instability in these tumor types.

Meanwhile, the researchers observed that, compared with primary cancers, metastatic cancers only have a moderate increase in tumor mutation burden (TMB), including single-base substitutions (SBSs), double-base substitutions (DBSs) and indels (IDs). This result indicates that TMB does not inevitably reflect the status of cancer progression, and the entire mutation spectrums are formed before or during primary cancer progression. Further comparison of mutational process activity revealed the existence of exogenous (such as exposure to platinum or radiation-based treatments) and endogenous (such as increase in APOBEC and SBS1 mutation burden) mutational processes that generate TMB differences. By investigating the divergence of SBS1 mutation burden in detail, the authors revealed a highly cancer type-specific SBS1 mutation burden per age. The majority of tumor types showed a prominent enrichment of SBS1 mutations along with age in both primary and metastatic lesions, however, 4 cancer types (thyroid, prostate, breast and clear cell renal cell cancers) only displayed increased SBS1 mutation burdens in metastatic cohorts in an age-independent manner.

Compared with TMB, an elevated frequency of structural variants (SVs) was noticed in most tumor types included in metastatic cohorts. The underlying genomic instability signatures linked to this phenomenon are TP53 alterations and genome ploidy, which exhibited significant pan-tumor correlations with duplications and deletions, thus likely to play an essential role in SV increase in metastatic cancers. In addition, the researchers found a modest overall increase of driver gene alterations in patients with metastatic cancers. The majority of genetic drivers enriched by cancer metastasis were tumor-type specific, including some well-known driver genes related to resistance to anticancer treatments, whereas 3 genetic drivers (TP53, CDKN2A and TERT) exhibited significant enrichment in metastatic cohorts across various cancer types, suggesting that changes in such genes may promote invasiveness via interfering with tumorigenesis hallmarks of pan-cancer. When comparing therapeutically actionable variants for each type of cancer, the authors found that the metastatic cohort had an overall higher proportion of patients with such variants, although the distribution was highly cancer type specific.

The presence of treatment-resistant genetic drivers in advanced cancers led the authors to identify therapeutically enriched drivers (TEDs) that were either treatment enriched or exclusively found in a treatment-specific and cancer type-specific manner. Ultimately, 61 TEDs related to 33 therapeutic groups were identified, and most top hits were well-established resistant drivers to anticancer treatments. Notably, TP53 alterations were found to be frequently relevant with multiple treatment resistances, indicating such variants are potential predictive markers for augmented cancer plasticity and aggressiveness instead of the resistance mechanism for a specific tumor type. Overall, TEDs could be found in 53% of patients in metastatic cohort. After excluding TEDs, the raw difference between primary and metastatic tumors will be reduced by 36% in the number of drivers per sample, suggesting that a significant portion of the metastatic-enriched drivers are very likely linked to anticancer treatment resistance.

Taken together, this study confirmed previous findings observed in certain cancer types and provided novel biological insights into the unique characteristics of metastatic cancers and their genomic differences with primary tumors, such as low intratumor heterogeneity, high genomic instability and elevated SVs. Nevertheless, the extent of genetic differences between metastatic and primary cancers varied significantly across cancer types and was affected by anticancer treatment exposure. Among the 23 tumor types, breast, clear cell renal cell, thyroid, prostate and pancreatic neuroendocrine cancers showed strong transformation in genomic landscape in advanced stages. In addition, this study provides a valuable resource for further investigating other aspects of cancer progression, such as the study on genetic immune escape alterations between metastatic and primary cancers conducted also by Edwin Cuppen’s group recently.^5^ Despite the aforementioned contributions, this study was also limited by the usage of distinct laboratory workups and sequencing procedures for the tumor samples from different datasets and the finite sample sizes of some cancer cohorts. Therefore, it is important to enlarge cancer cohorts and further uniform sample collation and sequencing processes to facilitate and validate the present understanding of cancer development. Moreover, genomic alterations are unable to fully elucidate cancer metastasis and resistance, and using the information from tumor microenvironment and additional cancer omics will also be essential for further anatomizing and better understanding the underlying mechanisms.

## References

[1] Martínez-Jiménez F (2023). Pan-cancer whole-genome comparison of primary and metastatic solid tumours. Nature.

[2] Hanahan D (2022). Hallmarks of cancer: new dimensions. Cancer Discov..

[3] Lambert AW, Pattabiraman DR, Weinberg RA (2017). Emerging biological principles of metastasis. Cell.

[4] Birkbak NJ, McGranahan N (2020). Cancer genome evolutionary trajectories in metastasis. Cancer Cell.

[5] Martínez-Jiménez F (2023). Genetic immune escape landscape in primary and metastatic cancer. Nat. Genet..

